# Reward Network Activations of Win Versus Loss in a Monetary Gambling Task

**DOI:** 10.3390/bs15080994

**Published:** 2025-07-22

**Authors:** Chella Kamarajan, Babak A. Ardekani, Ashwini K. Pandey, Gayathri Pandey, Sivan Kinreich, Weipeng Kuang, Jacquelyn L. Meyers, Bernice Porjesz

**Affiliations:** 1 Henri Begleiter Neurodynamics Laboratory, Department of Psychiatry and Behavioral Science, SUNY Downstate Health Sciences University, Brooklyn, NY 11203, USA; 2 Center for Biomedical Imaging and Neuromodulation, Nathan Kline Institute for Psychiatric Research, Orangeburg, NY 10962, USA; 3 Department of Psychiatry, NYU School of Medicine, New York, NY 10016, USA

**Keywords:** reward processing, monetary gambling task, reward network, functional MRI, impulsivity, neuropsychological tests, gambling performance

## Abstract

Reward processing is a vital function for health and survival and is impaired in various psychiatric and neurological disorders. Using a monetary gambling task, the current study aims to elucidate neural substrates in the reward network underlying the evaluation of win versus loss outcomes and their association with behavioral characteristics, such as impulsivity and task performance, and neuropsychological functioning. Functional MRI was recorded in thirty healthy, male community volunteers (mean age = 27.4 years) while they performed a monetary gambling task in which they bet with either 10 or 50 tokens and received feedback on whether they won or lost the bet amount. Results showed that a set of key brain structures in the reward network, including the putamen, caudate nucleus, superior and inferior parietal lobule, angular gyrus, and Rolandic operculum, had greater blood oxygenation level-dependent (BOLD) signals during win relative to loss trials, and the BOLD signals in most of these regions were highly correlated with one another. Furthermore, exploratory bivariate analyses between these reward-related regions and behavioral and neuropsychological domains showed significant correlations with moderate effect sizes, including (i) negative correlations between non-planning impulsivity and activations in the putamen and caudate regions, (ii) positive correlations between risky bets and right putamen activation, (iii) negative correlations between safer bets and right putamen activation, (iv) a negative correlation between short-term memory capacity and right putamen activity, and (v) a negative correlation between poor planning skills and left inferior occipital cortex activation. These findings contribute to our understanding of the neural underpinnings of monetary reward processing and their relationships to aspects of behavior and cognitive function. Future studies may confirm these findings with larger samples of healthy controls and extend these findings by investigating various clinical groups with impaired reward processing.

## 1. Introduction

Reward processing is a key neurocognitive function essential for survival in most species. Understanding the mechanisms of reward processing is crucial, as they are fundamental to human cognition and behavior, including motivation, learning, and decision-making (O’Doherty et al., 2017). Furthermore, dysfunction in reward processing is linked to several psychiatric disorders, such as addiction, depression, and obesity (Admon & Pizzagalli, 2015; Garcia-Garcia et al., 2014; Zald & Treadway, 2017), making it critical to elucidate the neural substrates and behavioral correlates of reward processing. While primary rewards (e.g., food, sex) and secondary rewards (e.g., money, tokens, and verbal reinforcements such as appreciation) (OpenStax & Lumen Learning, 2024) are important, in humans, the utmost importance is placed on monetary rewards, as they can buy most of the other rewards (Medvedev et al., 2024). Most human studies investigating reward processing have used monetary incentives as a proxy for primary rewards (Dichter et al., 2012). Furthermore, neuroimaging studies have indicated that primary rewards may evoke similar neural responses in humans in response to more abstract, or secondary, rewards such as monetary incentives (Ernst et al., 2004; Zald et al., 2004). Studies have also shown that gambling tasks are better suited to examining the neural substrates of monetary reward processing in humans than other paradigms (Bentivegna et al., 2024).

Over the decades, studies on animals and humans have employed various neuroimaging methods to elucidate the neural substrates of reward processing. In particular, many studies have used functional MRI (fMRI) to elucidate brain structures that are related to aspects of reward processing (Arsalidou et al., 2020; Flannery et al., 2020; Liu et al., 2011; McClure et al., 2004; Richards et al., 2013; Tomasi & Volkow, 2014; K. S. Wang et al., 2016). These studies have identified a set of subcortical (i.e., ventral tegmental area, nucleus accumbens, putamen, caudate, pallidum, amygdala, thalamus, and hippocampus) and cortical regions (i.e., insula, parahippocampal gyrus, cingulum, orbitofrontal cortex, angular gyrus, superior parietal lobule, inferior parietal lobule, and dorsolateral prefrontal cortex) that are activated during reward processing (Kamarajan et al., 2022). Studies have also found that individual variability in behavioral characteristics can affect reward processing and its neural substrates (Bentivegna et al., 2024; Jiang et al., 2024; Kamarajan et al., 2010; Medvedev et al., 2024). More specifically, psychological characteristics (e.g., impulsivity) (Bialaszek et al., 2015; Kamarajan et al., 2010), task performance (e.g., opting for risky vs. safer choices in tasks assessing reward processing) (Kamarajan et al., 2020; Kamarajan et al., 2010), and neuropsychological variables (e.g., executive functions, memory, etc.) (Kamarajan et al., 2020; Kamarajan et al., 2010) can modulate monetary reward processing. However, only a few studies have examined the associations of psychological, behavioral, and neuropsychological characteristics with the neural substrates of reward processing.

Impulsivity, a key factor known to modulate the reward processing mechanism (Beck et al., 2009; Diekhof et al., 2012), has three core aspects: (1) acting on the spur of the moment (motor impulsivity), (2) not focusing on the task at hand (attentional or cognitive impulsivity), and (3) lack of planning without adequate thinking (non-planning impulsivity) (Moeller et al., 2001; Patton et al., 1995). A previous fMRI study reported that individual differences in impulsivity accounted for variations in the reward-related blood oxygenation level-dependent (BOLD) response in the ventral striatum and the orbitofrontal cortex (Hahn et al., 2009). Furthermore, decreased activation of the ventral striatum and anterior cingulate during reward processing was shown to correlate with high impulsivity in those with alcohol use disorder (Beck et al., 2009). These findings emphasize the need to confirm the association between impulsivity and BOLD activation of specific brain structures during reward processing, particularly while evaluating monetary outcomes.

fMRI studies have identified specific brain regions associated with task-related behaviors. For example, putamen activity was shown to be associated with the stimulus–action–reward association (Haruno & Kawato, 2006) and with reward sensitivity (Mizuno et al., 2016), while midbrain activation was linked to efficiency in task performance (Krebs et al., 2012). However, the association between brain activation and participants’ performance style or strategies during monetary reward processing, such as choosing risky bets against safer options after previous losses, has not been adequately studied. Therefore, one of the aims of the current fMRI study is to examine this association during a monetary gambling task.

Lastly, studies have reported associations between cognitive abilities measured with neuropsychological tests and neural activation in specific brain structures during reward processing. For example, the basal ganglia structures, particularly the putamen, were found to modulate working memory in a delayed-response task of a reward paradigm (Yu et al., 2013), while putamen activity was associated with cognitive functions in general (Sefcsik et al., 2009) and learning and memory in particular (Muehlberg et al., 2024; Shu et al., 2009). Furthermore, executive functions such as planning and problem-solving may also be inherently associated with reward processing and related neural activation (Rovelli & Allegretta, 2023). Although brain–behavior associations in the context of reward processing have been examined by some studies in an isolated fashion, no study has investigated multiple behavioral and neuropsychological domains in the same participants.

Therefore, the current study aims to understand associations among the brain regions involved in reward processing networks and elucidate the neural substrates underlying the evaluation of win versus loss outcomes using a monetary gambling task in a group of healthy participants. Furthermore, the current study will investigate possible associations of activations of brain reward structures with key aspects of behavior and cognitive function (impulsivity, task performance, and neuropsychological measures).

## 2. Materials and Methods

### 2.1. Sample

The sample consisted of 30 healthy, young male participants (ages 19–38, mean = 27.4 years), who were recruited from the community. The exclusion criteria were (i) a diagnosis of a major psychiatric disorder or substance use disorders, (ii) a hearing/visual impairment, (iii) a history of head injury, and (iv) cognitive deficits or a score of <24 on the mini-mental state examination (MMSE). Most of the participants (28 out of 30) in the study were right-handed, and their education ranged from 12 to 20 years, with a mean of 15.8 years. Behavioral and neuropsychological data were collected at the SUNY Downstate Health Sciences University. The structural and functional MRI data were acquired at Nathan Kline Institute for Psychiatric Research and New York University. Informed consent was obtained from the participants, and the research protocol was approved by the Institutional Review Boards of all centers.

### 2.2. The Monetary Gambling Task (MGT)

**Figure 1:**
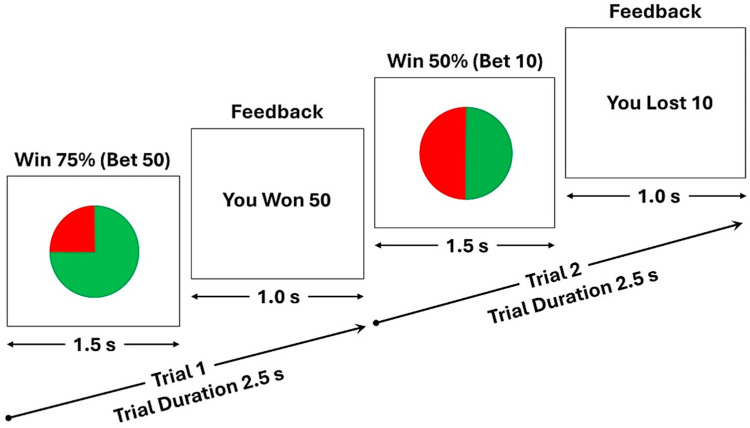
The schematic diagram of the monetary gambling task, showing two random trials: (i) a trial showing a pie stimulus (duration = 1.5 s) with a winning chance of 75%, for which a participant bet with 50 tokens and won the bet amount, as displayed in the feedback stimulus (duration = 1.0 s) (Trial 1); and (ii) another trial showing the pie stimulus with a winning chance of 50%, for which the participant bet with 10 tokens and lost the bet amount, as displayed in the feedback stimulus (Trial 2). The task consisted of 240 trials, and the length of each trial was 2.5 s.

The Monetary Gambling Task (MGT), as illustrated in Figure 1, consisted of 240 trials. The duration of each trial was 2.5 s, and it took about 10 min to complete the task. Each trial consisted of two stimulus presentations: (a) a pie stimulus presenting the chance of winning (75%, 50%, or 25%) for a duration of 1.5 s, during which the participant was instructed to select a bet amount of either 10 tokens, by pressing button 1, or 50 tokens, by pressing button 2, on a button response unit with their right hand; and (b) a feedback stimulus for a duration of 1.0 s, with a text indicating whether the participant had won or lost the bet amount. Thus, six types of trials were presented randomly, irrespective of the bet amount (see Table 1): (1) chance of winning = 50%; outcome = win; number of trials = 40; (2) chance of winning = 50%; outcome = loss; number of trials = 40; (3) chance of winning = 75%; outcome = win; number of trials = 60; (4) chance of winning = 75%; outcome = loss; number of trials = 20; (5) chance of winning = 25%; outcome = win; number of trials = 20; and (6) chance of winning = 25%; outcome = loss; number of trials = 60. The participants were not made aware of the trial types and sequence. If the participant failed to make a bet within 1.5 s after the presentation of the pie stimulus, a feedback stimulus “No bet made!” would appear on the screen. The total amount won or lost by the participant was displayed at the end of the task. The participants were informed that the final net tokens won would be converted to a dollar amount (e.g., 500 tokens = USD 5) and paid to them at the end of the session, although the net amount lost was not penalized.

### 2.3. Behavioral Scores Extracted from Task Performance

The list of behavioral scores that were computed for the participants based on their performance of the monetary gambling task is shown in Table 2. These scores included the following: (i) the total number of tokens won or lost at the end of the task [Net_Outcome]; (ii) the number of trials with a bet amount of 50 when the outcome/feedback was “loss” for the previous one, two, and three trials, respectively [Bet50_Prv1Loss, Bet50_Prv2Loss, and Bet50_Prv3Loss], suggesting potential risky behavior; (iii) the number of trials with a bet amount of 10 when the outcome/feedback was “loss” for the previous one, two, and three trials, respectively [Bet10_Prv1Loss, Bet10_Prv2Loss, and Bet10_Prv3Loss], suggesting potential safe behavior; (iv) the number of trials with a bet amount of 50 when the net outcome was “loss” for the previous two and three trials, respectively [Bet50_Prv2NetLoss and Bet50_Prv3NetLoss], indicating risky behavior; and (v) the number of trials with a bet amount of 10 when the net outcome was “loss” for the previous two and three trials, respectively [Bet10_Prv2NetLoss and Bet10_Prv3NetLoss], indicating safe behavior. The term “net outcome” for two or more consecutive trials refers to the resulting number of tokens lost or won during those trials. For instance, if the outcomes during the previous two trials were a win of 10 and a loss of 50, the net outcome for those two trials would be a loss of 40 tokens.

### 2.4. Neuroimaging Protocol

#### 2.4.1. Structural and Functional MRI Acquisition

Both structural and functional MRI data were collected at the Center for Biomedical Imaging and Neuromodulation, Nathan Kline Institute for Psychiatric Research, Orangeburg, NY, using a 3.0 Tesla Siemens Trio scanner (Erlangen, Germany). BOLD fMRI scans were acquired using a T2*-weighted gradient-echo single-shot echo-planar imaging (EPI) sequence with these acquisition parameters: number of slices = 36; voxel size = (2.5 × 2.5 × 3.5) mm^3^; FOV = 240 mm; TR = 2500 ms; TE = 30 ms; flip angle = 80°; parallelization factor = 2; acquisition time = 2.5 s per volume; and number of volumes = 240. The sequence was carefully optimized to minimize the effects of magnetic susceptibility inhomogeneities (such as distortions and signal dropouts), as well as the effects of mechanical vibrations, which elevate Nyquist ghosting levels. In addition, a magnetization-prepared rapid gradient-echo (MPRAGE) high-resolution three-dimensional T1-weighted structural sequence was collected to be used as an anatomical reference for the fMRI data as well as for the non-linear registration of imaging data between subjects. The sequence parameters for the MPRAGE were as follows: TR = 2500 ms; TE = 3.5 ms; TI = 1200 ms; flip angle = 8°; voxel size = 1 m × 1 m × 1 m; matrix size = 256 × 256 × 192; FOV = 256 mm; and number of averages = 1.

#### 2.4.2. fMRI Preprocessing

The *3dvolreg* module of the Analysis of Functional NeuroImages (AFNI) software package (version AFNI_18.1.09) (Cox, 1996) was used to detect and correct participants’ motion in the fMRI sequence. Accuracy of this motion detection method has been validated in a separate study (Ardekani et al., 2001). The procedure yielded a motion-corrected 4D image sequence of size 96 × 96 × 36 × 240 as well as six estimated rigid-body motion time series (three translations and three rotations). These time series were used as nuisance covariates in the general linear model (GLM) used for activation detection, as described below.

The 240 motion-corrected EPI volumes were averaged. An intensity threshold was applied to the average volume to obtain a brain mask for each participant. Subsequent voxel-wise analyses were confined to the brain mask voxels. A principal component analysis (PCA) was performed on the motion-corrected fMRI sequence. The first principal component capturing the greatest amount of variance in the data was also used as a nuisance covariate in the GLM.

#### 2.4.3. Subject-Level BOLD Response: Activation Detection Between Win and Loss

For each participant in the study, we separately performed a voxel-wise fitting of the BOLD signal to a GLM:

(1) $$y_{v}=X\beta_{v}+e_{v}$$

In this model, $y_{v}$ is the n×1 vector of BOLD signal observations after motion correction at voxel v (n=240); X is an n×p design matrix, as described below; $\beta_{v}$ is the p×1 vector of unknown parameters; and $e_{v}$ is an n×1 vector of random noise.

In our analysis, the number of columns of the design matrix p equaled 11. The first column was the eigenvector corresponding to the largest eigenvalue obtained by the PCA of the motion-corrected fMRI sequence across all brain voxels. As mentioned in the previous section, this was included as a nuisance covariate. The next six columns of X were occupied by the six estimated rigid-body motion parameters obtained using *3dvolreg*. As mentioned in the previous section, these were also included in the design matrix as nuisance covariates. Finally, columns 8–11 of X were designed to model the hemodynamic response during the MGT task, as described below.

We modeled the hemodynamic response to each task condition (win and loss) with the gamma hemodynamic response function (HRF) described in Ardekani et al. (2002) and Rangaswamy et al. (2004). The model thus obtained comprised the last two columns of X. Specifically, if we represent the HRF by h, the last two columns of X were given by $h*z_{j}$, where ∗ represents the convolution operation. Alternatively, we can represent the n×2 sub-matrix comprised of the last two columns of the design matrix as h∗Z, where it is understood that the convolution operation is applied to the columns of Z.

Let G represent the pseudo-inverse of the p×p matrix $X^{t}X$. The parameters of the GLM are estimated as ${{\hat{\beta }}_{v}=GX}^{t}y_{v}$. Since we were interested in the brain networks that are activated during successful inhibition of a motor response to a visual stimulus, we looked for voxels for which the contrast $C^{t}{\hat{\beta }}_{v}$ was large relative to the amplitude of the random variations, where $C^{t}={\left[\begin{array}{cccccc} 0 & \cdots & -1 & 1 & 0 & 0 \end{array}\right]}^{t}$. A variable that captures this notion is given by the following:

(2) $$t_{v}=\frac{C^{t}{\hat{\beta }}_{v}}{\sqrt{{\hat{\sigma }}^{2}C^{t}GC}}$$

where ${\hat{\sigma }}^{2}=(y^{t}y-y^{t}X^{t}GXy)/df$ and df=n−1−rank(G).

Note that under certain normality and independence conditions, $t_{v}$ would have a Student’s *t* distribution with df degrees of freedom. However, we will not make such assumptions and treat $t_{v}$ simply as a variable designed to indicate the level of activation at voxel v. By computing this index for all voxels, we obtained an activation map for each of the participants in our study. The activation map identified using this method was further subjected to refinement using a Sparse Principal Component Analysis (sPCA) method (Sjöstrand et al., 2006; Zou et al., 2006), which is described in Section 2.4.5. A similar method was implemented in our previous work (Pandey et al., 2022).

#### 2.4.4. Group-Level Image Processing of the fMRI Data

Processing of the imaging data included the following stages. Within each subject, the MPRAGE and fMRI volumes were registered using the intra-subject inter-modality linear registration module (Ardekani et al., 1995) of the Automatic Registration Toolbox (ART; www.nitrc.org/projects/art (accessed on 11 January 2018)). The *brainwash* program within the ART toolbox was used for skull-stripping the MPRAGE volumes. To correct for small subject motion during fMRI acquisitions, motion detection and correction were performed using the *3dvolreg* module of the AFNI software package (version AFNI_18.1.09) (Cox, 1996). To correct for the geometric distortions of the fMRI images due to magnetic susceptibility differences in the head, particularly at brain/air interfaces, we used the non-linear registration module of the ART (Ardekani et al., 2005). The skull-stripped MPRAGE images from all subjects were non-linearly registered to a study-specific population template using ART’s non-linear registration algorithm *3dwarper*, which is one of the most accurate inter-subject registration methods available (Klein et al., 2009). The population template was formed using an iterative method (Joshi et al., 2004). The motion-corrected fMRI time series were detrended using PCA (Koay et al., 2006). Finally, fMRI data from all subjects were normalized to a standard space using the image registration steps outlined above, which were mathematically combined into a single transformation and used in resampling the fMRI data.

#### 2.4.5. Refinement of BOLD Activation Clusters for the Win–Loss Contrast

After creating the activation maps using GLM as described in Section 2.4.3, we used a Sparse Principal Component Analysis (sPCA) method (Sjöstrand et al., 2006; Zou et al., 2006) to further refine the activation clusters from the data matrix containing the 7050 voxels that were detected to be activated during the task conditions. sPCA can stringently reduce the non-zero voxels that are not relevant to the activation clusters based on a specific threshold. While the components from sPCA have a natural ordering according to their variance, similar to regular PCA, sPCA performed better in separating the noise from the signal and was found to be more flexible, less committed, and easier to interpret (Sjöstrand et al., 2006; Tibshirani, 1996). sPCA extracts a relatively minimal number of non-zero components by using the LASSO regression technique (Tibshirani, 1996), which drives some loadings to exactly zero and adjusts the other components to approximate the properties of PCA (Sjöstrand et al., 2006).

We used the top clusters with a size of 100 voxels or more for statistical analyses (see Table 3). The activation value for each cluster was determined by taking the mean from all of its voxels. The anatomical labels for the clusters, based on the MNI coordinates of their centroids, were extracted using the automated anatomical labeling (AAL) method (Rolls et al., 2020), as provided by the R-package *label4MRI* (https://github.com/yunshiuan/label4MRI (accessed on 11 January 2018)).

### 2.5. Assessment of Impulsivity

The participants were administered the Barratt Impulsiveness Scale—Version 11 (BIS-11) (Patton et al., 1995), a 30-item self-administered tool that assesses motor impulsivity (BIS_MI), non-planning (BIS_NP), attentional impulsivity (BIS_AI), and the total score (BIS_Tot). BIS-11 has been widely used to assess impulsivity and its biological, psychological, and behavioral correlates (Reise et al., 2013). Attentional impulsivity refers to an inability to focus attention or concentrate on the job at hand, motor impulsivity is the tendency to act on the spur of the moment without thinking, while non-planning impulsivity is conceptualized as a lack of forethought or planning when executing a task (Stanford et al., 2009). The data obtained for each subscale and the total score of the Barratt Impulsiveness Scale are shown in Table 3.

### 2.6. Neuropsychological Assessment

Computerized adaptations of the Tower of London test (TOL) (Shallice, 1982) and the visual span test (VST) (Berch et al., 1998; Milner, 1971) were administered using the Colorado assessment tests for cognitive and neuropsychological assessment (Davis & Keller, 2002), as described previously (Pandey et al., 2018). Details of these tests are summarized below.

#### 2.6.1. Tower of London Test (TOL)

The planning and problem-solving ability of the executive functions was assessed using the TOL, in which participants solved a set of puzzles with graded difficulty levels by arranging color beads one at a time from a starting position to a desired goal position in as few moves as possible. The test consisted of three puzzle types with three, four, and five colored beads placed on the same number of pegs, with seven problems/trials per type and a total of twenty-one trials. The five performance measures from the summation of all puzzle types used in the analysis and their codes were as follows: (i) excess moves made (additional moves beyond the minimum moves required to solve the puzzle) [TOL_ExcMovMade]; (ii) the average pickup time (initial thinking/planning time spent until picking up the first bead to solve the puzzle) [TOL_AvgPicTime]; (iii) the average total time (total thinking/planning time to solve the problem in each puzzle type) [TOL_AvgTotTime]; (iv) the total trial time (total performance/execution time spent on all trials within each puzzle type) [TOL_TotTrlTime]; and (v) the average trial time (mean performance/execution time across trials per puzzle type) [TOL_AvgTrlTime]. The performance scores from the TOL test are shown in Table 4.

#### 2.6.2. Visual Span Test (VST)

The VST was used to assess the visuospatial memory span from the forward condition and working memory from the backward condition. In this test, 8 randomly arranged squares were displayed on the screen, and 2–8 squares flashed in a predetermined sequence, depending on the span level being assessed. Each span level was administered twice, with a total of 14 trials in each condition. During the forward condition, subjects were required to repeat the sequence in the same order via mouse clicks on the squares. In the backward condition, subjects were required to repeat the sequence in reverse order (starting from the last square). The four performance measures collected during the forward and backward conditions (with a total of eight scores and their codes) were as follows: (i) total correct scores (total number of correctly performed trials) [VST_TotCor_Fw and VST_TotCor_Bw]; (ii) span (maximum sequence length achieved) [VST_Span_Fw and VST_Span_Bw]; (iii) total average time (sum of the mean time taken across all trials performed) [VST_TotAvgTime_Fw and VST_TotAvgTime_Bw]; and (iv) total correct average time (sum of the mean time taken across all trials correctly performed) [VST_TotCorAvgTime_Fw and VST_TotCorAvgTime_Bw]. The performance scores from the VST are shown in Table 5.

### 2.7. Statistical Analyses

All statistical analyses were performed using the R packages (R Core Team, 2024). Pearson bivariate correlations were performed to identify significant relationships across the fMRI activation clusters. We also performed an exploratory correlational analysis to test the associations between the fMRI activation clusters and behavioral/cognitive variables such as demographic variables (age and education), impulsivity scores (BIS_NP, BIS_MI, BIS_AI, and BIS_Tot), gambling task performance measures (Net_Outcome, Bet50_Prv1Loss, Bet10_Prv1Loss, Bet50_Prv2Loss, Bet10_Prv2Loss, Bet50_Prv3Loss, Bet10_Prv3Loss, Bet50_Prv2NetLoss, Bet10_Prv2NetLoss, Bet50_Prv3NetLoss, and Bet10_Prv3NetLoss), and neuropsychological scores (TOL_ExcMovMade_All, TOL_AvgPicTime_All, TOL_AvgTotTime_All, TOL_TotTrlTime_All, TOL_AvgTrlTime_All, VST_TotCor_Fw, VST_Span_Fw, VST_TotAvgTime_Fw, VST_TotCorAvgTime_Fw, VST_TotCor_Bw, VST_Span_Bw, VST_TotAvgTime_Bw, and VST_TotCorAvgTime_Bw). Descriptions of variables in each domain are available in the previous sections. Significant correlations were highlighted. However, in order to avoid a Type II error (Streiner & Norman, 2011; Sullivan & Feinn, 2012) due to the small sample size, multiple testing corrections were not performed for the exploratory correlational analysis, and the strength of association was determined based on the magnitude of the correlation coefficient as a metric of effect size (Hemphill, 2003). However, it is possible that there is a potential risk of Type 1 errors due to multiple testing despite using the size of the correlation coefficient as a metric for effect size; therefore, the results and interpretation would be only preliminary.

## 3. Results

### 3.1. The fMRI Activation Clusters for the Win–Loss Contrast

**Figure 2:**
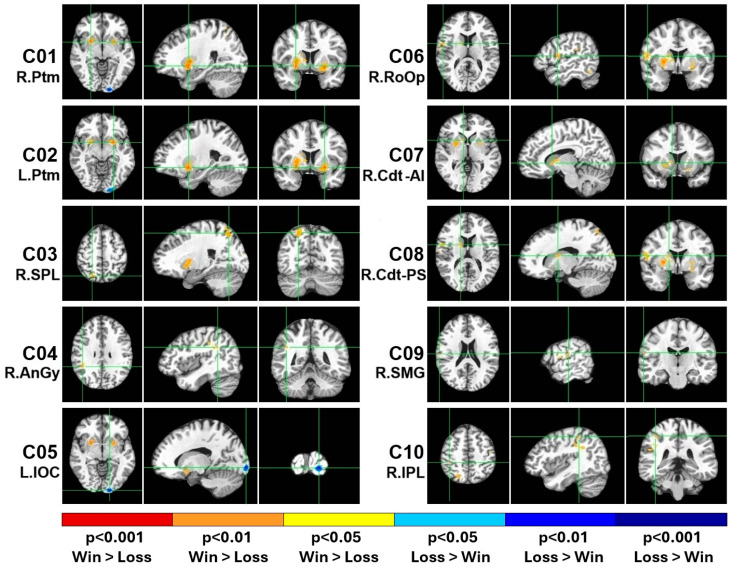
The 10 fMRI activation clusters (C01–C10) with 100 or more voxels that were extracted from the win–loss contrast of the monetary gambling task. The centroid of each cluster is shown with green crosshair lines for the axial (left panels), sagittal (middle panels), and coronal (right panels) views. The activated voxels are highlighted in orange/red (win > loss) or cyan/blue (loss > win) based on their values as shown in the color scale. Note that the long labels of the clusters are shown in Table 6.

As shown in Table 3 and Figure 2, the BOLD activation difference between the gambling outcomes (win–loss) elicited 10 regions (i.e., clusters), with 100 or more voxels showing activations: (i) the right putamen, (ii) the left putamen, (iii) the right superior parietal lobule, (iv) the right angular gyrus, (v) the left inferior occipital cortex, (vi) the right Rolandic operculum, (vii) the right caudate (anterior–inferior), (viii) the right caudate (posterior–superior), (ix) the right supramarginal gyrus, and (x) the right inferior parietal lobule. Eight of the ten clusters were on the right side, with only two clusters on the left, namely, the left putamen and left inferior occipital cortex. Nine of the ten clusters showed higher activation during the win relative to the loss condition (orange/red blobs in Figure 2), with only the left inferior occipital cortex (cluster # 5) showing higher activation during loss (cyan/blue blobs in Figure 2). Overall, eight clusters represented the right hemisphere, and two clusters were in the left hemisphere. The putamen showed bilateral activations, represented by clusters 1 and 2. The right caudate was represented by two separate clusters in different locations (i.e., cluster 7 was at the anterior–inferior location, and cluster 8 was at the posterior–superior location). Interestingly, three of the cortical clusters (i.e., clusters 4, 9, and 10) represented the inferior parietal lobule, and two of them (clusters 9 and 10) represented the same Brodmann area (BA 40), although cluster 10 (R. IPL) was more medial, posterior, and superior to the anatomical location of cluster 9 (R. SMG).

**Table 6:** The fMRI activation clusters for the win–loss contrast, which had 100 or more voxels. The number of voxels, anatomical region, Brodmann area, MNI coordinates, along with the mean, SD, and SE values of cluster average data obtained from each participant, are shown for each cluster.

#	Size	Anatomical Region	Code	Direction	BA	MNI	Mean	SD	SE
1	1781	R. Putamen	R. Ptm	Win > Loss	49	27,5,−6	62.87	63.99	11.68
2	1426	L. Putamen	L. Ptm	Win > Loss	49	−24,5,−9	67.61	62.27	11.37
3	878	R. Superior Parietal Lobule	R. SPL	Win > Loss	7	23,−68,56	80.96	120.22	21.95
4	663	R. Angular Gyrus	R. AnGy	Win > Loss	39	44,−47,30	61.00	64.65	11.80
5	640	L. Inferior Occipital Cortex	L. IOC	Loss > Win	18	−15,−100,−6	−97.03	77.14	14.08
6	444	R. Rolandic Operculum	R. RoOp	Win > Loss	6	56,2,12	55.53	66.63	12.16
7	333	R. Caudate (anterior–inferior)	R. Cdt (A-I)	Win > Loss	48	11,12,0	53.03	81.01	14.79
8	239	R. Caudate (posterior–superior)	R. Cdt (P-S)	Win > Loss	48	17,1,14	54.47	100.90	18.42
9	100	R. Supramarginal Gyrus	R. SMG	Win > Loss	40	63,−18,20	75.13	97.02	17.71
10	100	R. Inferior Parietal Lobule	R. IPL	Win > Loss	40	42,−37,51	63.64	109.55	20.00

# Cluster Number; Code = Short name of the anatomical region; R = Right Hemisphere; L = Left Hemisphere; BA = Brodmann Area; MNI = MNI coordinates; Min = Minimum; Max = Maximum; SD = Standard Deviation; SE = Standard Error.

**Figure 3:**
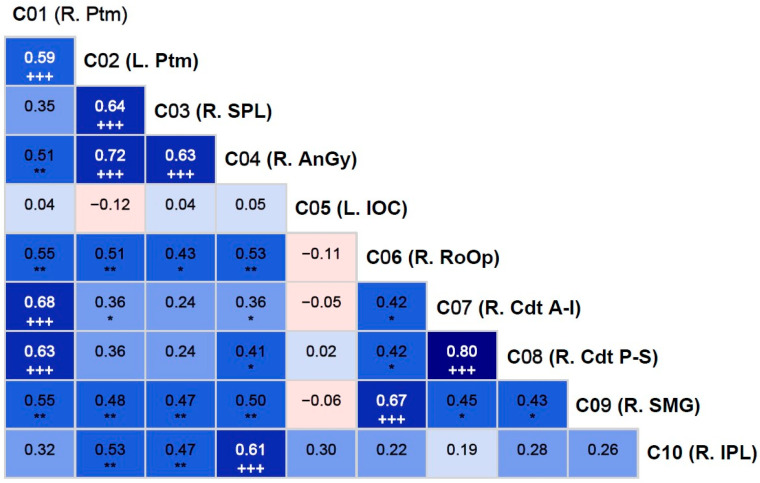
Pearson bivariate correlations among the fMRI activation clusters (C01–C10). The correlation coefficient (number) and the level of significance (asterisks and plus signs) are provided within each cell. The asterisks (* *p* < 0.05; ** *p* < 0.01) represent significance before the Bonferroni corrections, while the plus signs (+++) indicate those that survived Bonferroni correction. The cyan/blue shades represent positive correlations, and the pink shades indicate negative correlations.

Correlations among the fMRI activation clusters are shown in Figure 3. These inter-cluster correlations are between the items/variables within the same domain (i.e., brain regions), and it is possible that there could be collinearity across the clusters. Therefore, these correlations require a rigorous correction procedure for multiple testing to avoid any Type I errors. All clusters except cluster 5 (L. IOC) showed significant positive correlations with other clusters, even after correcting for multiple testing. Bonferroni-adjusted significant correlations were between (i) cluster 1 (R. Ptm) and cluster 2 (L. Ptm); (ii) cluster 1 (R. Ptm) and cluster 7 (R. Cdt A-I); (iii) cluster 1 (R. Ptm) and cluster 8 (R. Cdt P-S); (iv) cluster 2 (L. Ptm) and cluster 3 (R. SPL); (v) cluster 2 (L. Ptm) and cluster 4 (R. AnGy); (vi) cluster 3 (R. SPL) and cluster 4 (R. AnGy); (vii) cluster 4 (R. AnGy) and cluster 10 (R. IPL); (viii) cluster 6 (R. RoOp) and cluster 9 (R. SMG); and (ix) cluster 7 (R. Cdt A-I) and cluster 8 (R. Cdt P-S). Overall, clusters 1, 2, and 4 had three significant correlations each, followed by clusters 3, 7, and 8, which had two correlations each, and clusters 6 and 9, which had a single correlation with each other.

### 3.2. Correlations Between the fMRI Activation Clusters and Other Variables

**Figure 4:**
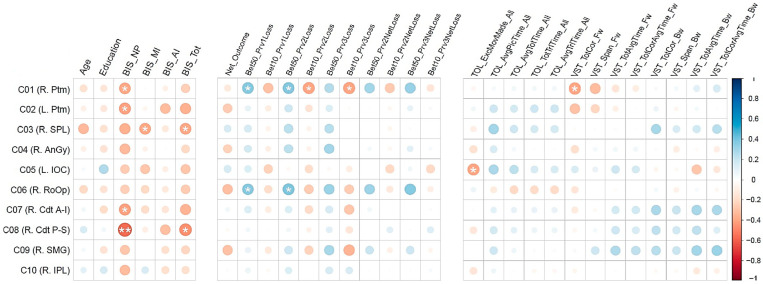
Pearson bivariate correlations between the fMRI activation clusters (C01-C10) and demographic variables (age and education), BIS impulsivity scores, gambling task performance measures, and neuropsychological scores from the TOL test and VST. The correlation values are represented by the size and color of the circles. The asterisks (* *p* < 0.05; ** *p* < 0.01) represent the level of significance before the Bonferroni corrections. The cyan/blue shades represent positive correlations, and the pink/red shades indicate negative correlations. Note that none of the correlations survived Bonferroni corrections, although some of the correlations do have moderate effect sizes.

Pearson bivariate correlations between the fMRI clusters (C01–C10) and other variable sets (demographic variables, impulsivity scores, task performance measures, and neuropsychological performance) are shown in Figure 4 and Table 4. While some correlations in each variable set (except in the demographic set) were significant and with moderate–high effect sizes ranging from 0.3617 to 0.5603 (Hemphill, 2003), none of these correlations survived multiple testing corrections due to the small sample size. When the sample size is small, as in our case, it may be better to rely on the effect size of the correlations rather than on multiple testing corrections of the significant correlations (Streiner & Norman, 2011; Sullivan & Feinn, 2012). Therefore, we have opted to identify significant correlations based on their effect sizes in order to avoid a Type II error (see Section 2.7) because the brain–behavior correlations (e.g., between activation clusters and measures of impulsivity, risk-taking, or cognitive performance) are across variables from different domains and are exploratory in nature. These significant correlations within each variable set are listed below:

*Impulsivity:*

(i) Negative correlation of BIS non-planning with fMRI activation cluster 1 (R. Ptm; r = −0.3844, *p* < 0.05), cluster 2 (L. Ptm; r = −0.4057, *p* < 0.05), cluster 7 (R. Cdt A-I; r = −0.4073, *p* < 0.05), and cluster 8 (R. Cdt P-S; r = −0.5603, *p* < 0.01);
(ii) Negative correlation of BIS motor impulsivity with cluster 3 (R. SPL; r = −0.3885, *p* < 0.05);
(iii) Negative correlation of BIS total impulsivity with cluster 3 (R. SPL; r = −0.3851, *p* < 0.05) and cluster 8 (R. Cdt P-S; r = −0.4504).

*Task Performance:*

(i) Positive correlations between the number of bets with 50 tokens after a loss during the previous trial with fMRI activation cluster 1 (R. Ptm; r = 0.3700, *p* < 0.05) and cluster 6 (R. RoOp; r = 0.3617, *p* < 0.05);
(ii) Positive correlations between the number of bets with 50 tokens after two consecutive losses during previous trials with fMRI activation cluster 1 (R. Ptm; r = 0.3754, *p* < 0.05) and cluster 6 (R. RoOp; r = 0.3896, *p* < 0.05);
(iii) Negative correlations of fMRI activation cluster 1 (R. Ptm) with the number of bets with 10 tokens after consecutively losing during the previous two trials (r = −0.3903, *p* < 0.05) as well as with the number of bets with 10 tokens after consecutively losing during the previous three trials (r = −0.3943, *p* < 0.05).

## 4. Discussion

The current study aimed to elucidate the neural substrates of monetary reward outcomes and their association with behavioral and cognitive features. Ten BOLD activation clusters were identified for the win–loss contrast (see Table 3 and Figure 2), which included the bilateral putamen, the right caudate nucleus, the right superior and inferior parietal lobule, the right angular gyrus, and the right Rolandic operculum. These anatomical regions showed greater activation during the win condition relative to the loss condition. It was found that all clusters except cluster 5 (left inferior occipital cortex) showed significant positive correlations with other clusters, suggesting that these brain regions were activated together during reward processing. Furthermore, exploratory bivariate correlations with moderate effect sizes suggested possible associations between these reward regions and some behavioral and cognitive characteristics, including (i) negative correlations between non-planning impulsivity and activations in the putamen and caudate regions, (ii) positive correlations between risky bets and right putamen activation, (iii) negative correlations between safer bets and right putamen activation, (iv) a negative correlation between short-term memory capacity and right putamen activity, and (v) a negative correlation between poor planning skills and left inferior occipital cortex activation.

### 4.1. Neural Substrates of the Win–Loss Contrast

#### 4.1.1. The Regions Activated During Reward Processing

The current study identified ten BOLD activation clusters for the win–loss contrast (see Table 3 and Figure 2): (i) the right putamen, (ii) the left putamen, (iii) the right superior parietal lobule, (iv) the right angular gyrus, (v) the left inferior occipital cortex, (vi) the right Rolandic operculum, (vii) the right caudate (anterior–inferior), (viii) the right caudate (posterior–superior), (ix) the right supramarginal gyrus, and (x) the right inferior parietal lobule. It is worth noting that most of these regions were part of the reward network, as reported by previous studies (Arsalidou et al., 2020; K. S. Wang et al., 2016). Our study identified brain structures in the dorsal striatum, such as the putamen (clusters 1 and 2) and the caudate nucleus (clusters 7 and 8), which are the core structures of the reward network (K. S. Wang et al., 2016). According to Arsalidou et al. (2020), a common reward processing circuit is composed of basal ganglia nuclei such as the caudate, putamen, and globus pallidus, and these nuclei represent a basic subcortical structure that subserves reward processes irrespective of the reward outcome type or contextual factors associated with the rewards. Anatomically, both the putamen and the caudate project to the globus pallidus, which in turn has projections to the thalamus (Ikemoto et al., 2015). Broadly, the mesocorticolimbic reward system includes dopaminergic projections from the ventral tegmental area to both the nucleus accumbens and the dorsal striatum (i.e., the caudate and putamen) as well as to the orbital frontal cortex (OFC), medial prefrontal cortex (mPFC), and amygdala (Nestler & Carlezon, 2006). Although our study did not implicate the ventral striatum (i.e., nucleus accumbens), the putamen and caudate structures are more involved in monetary reward processing than other basal ganglia structures (Arsalidou et al., 2020). However, it is also quite likely that the absence of ventral striatum (nucleus accumbens) activation in our study could be due to methodological issues, such as task timing, sensitivity to reward stimuli, etc., as other task conditions (e.g., bet amounts and probability conditions related to reward outcomes) were not analyzed in the current study.

In terms of laterality, eight of the ten clusters represented the right hemisphere, except the two left-hemisphere clusters. A meta-analysis of fMRI studies on reward processing indicated that monetary rewards activated all the basal ganglia nuclei bilaterally, with the exception of the lateral globus pallidus (Arsalidou et al., 2020). In our study, while the putamen was involved bilaterally, only the right caudate was implicated in the win–loss contrast, along with other right-hemisphere regions, such as the superior parietal lobule, angular gyrus, supramarginal gyrus, and Rolandic operculum. The superior parietal lobule is involved in visual attention, spatial perception, visuomotor functions, spatial reasoning, and visual working memory (J. Wang et al., 2015), which are important elements during the evaluation of rewards and risk while performing the visual monetary gambling task, as used in our study. On the other hand, regions of the inferior parietal lobule (cluster 10), such as the angular gyrus (cluster 4) and supramarginal gyrus (cluster 9), are known to be involved in language ability, future planning, problem-solving, calculations, and other complex mental operations (Numssen et al., 2021), some of which are essential while processing monetary reward stimuli. Moreover, other neuroimaging studies have implicated the inferior parietal lobule when evaluating the possible motor significance of sensory stimuli (Toni et al., 2001), perceptually based decisions, and prospective action judgment (Parsons et al., 1995), the functions that are also essential during the performance of gambling tasks. The right Rolandic operculum (cluster 6) was found to be associated with affective evaluation and depression (Sutoko et al., 2020), possibly with loss outcomes during the performance of a gambling task. Lastly, the left inferior occipital cortex (cluster 5; Brodmann area 18) showed higher activation during loss than during win outcomes. This secondary visual association cortex is known to be involved in the visual processing of color, motion, and depth perception (Kaas, 2017), functions that are practically imperative when performing a gambling task that contains the processing of visual stimuli presented on a computer screen. Furthermore, occipital cortex activation during the loss condition may represent motivated attention to affective or reward stimuli (Bradley et al., 2003), suggesting that attentional processing of visual stimuli at the occipital cortex could be modulated by reward signals (Chelazzi et al., 2013). Overall, most of the brain regions activated for the win–loss contrast in our study are key regions of the reward circuitry and are consistent with the previous findings on monetary reward processing.

#### 4.1.2. Correlations Across the fMRI Activation Clusters

One of the sub-aims of the study is to determine if the reward-related activation clusters are correlated or connected with one another. As shown in Figure 3, all clusters except cluster 5 (L. IOC) showed significant positive correlations with one or more other regions. Specifically, both the right and left putamen were correlated with each other, which is expected during monetary reward processing (Arsalidou et al., 2020). Furthermore, both clusters of the right caudate (clusters 7 and 8) were highly correlated with each other and with the right putamen (cluster 1), which is supported by the previous findings of bidirectional anatomical connectivity between the putamen and caudate nuclei (Arsalidou et al., 2020; Parent & Hazrati, 1995), suggesting a dynamic interplay between the caudate nucleus and the putamen in reward-related, instrumental behaviors (Brovelli et al., 2011). In addition, right superior parietal lobule (cluster 3) activation is correlated with that of the left putamen (cluster 2) and the right angular gyrus (cluster 4), possibly suggesting functional connectivity across these regions while processing potential monetary rewards (Jarbo & Verstynen, 2015). Similarly, the right angular gyrus (cluster 4; BA 39) was correlated with the right inferior parietal lobule (cluster 10; BA 40), indicating a strong functional link across these areas of multimodal regions responsible for visuospatial attention and other higher cognitive functions during visual tasks involving stimulus evaluation (Numssen et al., 2021). Lastly, the correlation between the right Rolandic operculum (cluster 6) and the right supramarginal gyrus (cluster 9) may represent an evaluation of the subjective emotional state associated with gambling outcomes, as these adjacent cortical regions are often related to the subjective evaluation of emotions (Sutoko et al., 2020) and the affective states (Silani et al., 2013), respectively.

### 4.2. Associations Between the Reward Regions and Behavioral Features

Exploratory bivariate correlations revealed several important and meaningful associations across the individual variables, with r-values ranging from 0.3617 to 0.5603, indicating moderate effect sizes (Figure 4 and Table 4). These associations are relevant and meaningful and may guide future studies in examining them systematically. Therefore, it is worth discussing these associations in a broader context. However, caution needs to be exercised, as these findings are only preliminary and exploratory, and they are also vulnerable to potential Type I errors, compromising the confidence of their interpretations or implications. Furthermore, the findings related to neural–behavioral associations may not imply directional causality, as this is only a cross-sectional explorative study. Therefore, the interpretations of these findings need not be understood as causative and directional.

#### 4.2.1. Associations Between the Reward Regions and Impulsivity

Exploratory bivariate correlations identified a few negative correlations between impulsivity and fMRI activation clusters (Figure 4 and Table 7), which include (i) non-planning impulsivity, with all four striatal structures, such as the bilateral putamen and right caudate (clusters 1, 2, 7, and 8); (ii) motor impulsivity, with the right superior parietal lobule (cluster 3); and (iii) total impulsivity, with the right superior parietal lobule (cluster 3) and with the right caudate (cluster 8). These findings indicate that higher impulsivity was associated with lower activation in those regions for the contrast of win–loss. In other words, those with heightened impulsivity showed either lower activation during win processing or higher activation during the loss condition, and vice versa may be true for those with lower impulsivity. While the theories of choice and decision-making posit that loss looms larger than gain in most individuals (Kahneman & Tversky, 1979), our findings indicate that sensitivity to loss is reflected more in those with higher impulsivity. Specifically, all four clusters representing core reward structures of the striatum were correlated with this non-planning impulsivity, a predisposition toward rapid, unplanned reactions to internal or external stimuli without regard to the negative consequences (Moeller et al., 2001), suggesting that impulsive people manifest an urge for immediate gratification, regardless of whether the immediate reward is certain or uncertain (Bialaszek et al., 2015). On the other hand, motor impulsivity and total impulsivity showed a negative association with the right superior parietal lobule, a region that is modulated by the reward amount and its probability while performing decision-making tasks (P. Wang et al., 2023). Although the total impulsivity score was also correlated with the right caudate (cluster 8), this was mostly contributed by the non-planning score. However, it should be noted that the correlation between brain activation and impulsivity may not imply causation, as this study is cross-sectional and exploratory.

#### 4.2.2. Associations Between the Reward Regions and Gambling Performance

With regard to associations between performance variables of the gambling task and the fMRI activation clusters, the exploratory analysis identified a few meaningful correlations (Figure 4 and Table 7). Interestingly, the right putamen (cluster 1) showed (i) positive correlations with risky choices, such as betting with 50 (a bigger amount) following a loss during the previous trial and previous two trials of the gambling task, and (ii) negative correlations with safer choices, such as betting with 10 (smaller amount) following a loss during the previous two and three trials. This finding of higher activation of the right putamen for risky bets and lower activation for safer or less risky bets suggests that the right putamen is modulated by how much is at stake and how much risk or loss is anticipated (i.e., loss sensitivity) during each trial. This finding is consistent with that of other studies that the putamen is associated with the stimulus–action–reward association (Haruno & Kawato, 2006) and low putamen activity is associated with poor reward sensitivity (Mizuno et al., 2016). Furthermore, risky choices (i.e., higher bets in the face of previous loss) were also positively correlated with the right Rolandic operculum (cluster 6), a cortical region associated with negative affective states such as apathy, depression, anxiety, and perceived stress (Sutoko et al., 2020), possibly representing a stressful state of anticipating a potential negative outcome during the gambling task. While our findings are very meaningful in the context of neural correlates underlying reward processing during a monetary gambling task, more studies with larger sample sizes are needed to further confirm and explain these preliminary findings. Moreover, the correlation between brain activation and gambling performance may not imply causation, as this study is cross-sectional and exploratory.

#### 4.2.3. Associations Between the Reward Regions and Neuropsychological Scores

The results from the current study also pointed to a couple of associations between neuropsychological variables and the fMRI activation clusters (Figure 4 and Table 7). First, a negative correlation was observed between right putamen activation and the total correct score of the total items correctly remembered during the visual span test, representing short-term memory capacity. This may indicate that individuals who had poor short-term memory capacity showed relatively high activation during the win condition (compared to the loss condition), or conversely, those with higher short-term memory capacity showed lower activation for the win condition (relative to the loss condition). In other words, putamen activation during reward processing varied based on the short-term memory capacity of the individuals. A previous study reported that the putamen may modulate working memory (Yu et al., 2013) during a delayed-response task that requires memory updating. The putamen was also found to modulate cognitive functions in general (Sefcsik et al., 2009) and learning and memory in particular (Muehlberg et al., 2024; Shu et al., 2009). It is possible that basal ganglia and medial temporal lobe memory systems work together in a complementary manner based on the task at hand (Packard & Knowlton, 2002). However, more studies are needed to confirm the exact role of the putamen under specific task conditions.

Furthermore, one of the fMRI activation clusters, the left inferior occipital cortex, was negatively correlated with the number of excess moves made (i.e., poor planning) during the Tower of London test, suggesting that higher activation of this brain region was associated with better cognitive planning. Although the inferior occipital cortex (Brodmann area 18), which is the secondary visual association cortex, does not have a specific role in the core aspect of reward processing per se, it is shown to be activated during visual–spatial tasks requiring visual attention to process and integrate various features of visual stimuli, including color, shape, texture, and brightness (Cavina-Pratesi et al., 2010), and to identify objects representing specific contexts and task demands (McGuire et al., 2022). Essentially, visual processing within the occipital cortex is a fundamental prerequisite for higher-level cognitive processes in both the gambling task (decision-making) and the TOL task (planning) to occur, as a heightened attentional or motivational response is required to these specific visual stimuli that represent the task demands. For example, studies have shown that in addition to prefrontal and subcortical activations, increased activity of the occipital cortex was observed during the performance of the TOL task (Baker et al., 1996; Rasmussen et al., 2006) as well as in response to visual gambling cues (Crockford et al., 2005; Goudriaan et al., 2010; Miedl et al., 2010), implying the role of the occipital cortex in visual attention (Kastner & Ungerleider, 2000) and visual working memory (Ungerleider et al., 1998) during higher cognitive processes. Lastly, it should be noted that the correlation between brain activation and neuropsychological performance may not imply causation, as this study is cross-sectional and exploratory.

#### 4.2.4. Clinical Implications

Empirical evidence supports reward network dysfunction in substance use disorders (Kalivas & Volkow, 2005) and other psychiatric disorders (Pujara & Koenigs, 2014). Problems with both impulsivity and reward processing underlie several psychiatric disorders (Swann et al., 2002), including substance use disorders (Allen et al., 1998; Coffey et al., 2003; Dawe et al., 2004; de Wit, 2009; Petry, 2001; Rubio et al., 2007), attention-deficit hyperactivity disorder (Andreou et al., 2015; Gomez, 2003), antisocial personality (Hesselbrock & Hesselbrock, 1992; Swann et al., 2009), conduct disorder (Castellanos-Ryan et al., 2011; Cherek & Lane, 1999), borderline personality disorder (Lawrence et al., 2010; van Reekum et al., 1994), eating disorder (Dawe & Loxton, 2004; Hege et al., 2015), and gambling addiction (Blaszczynski et al., 1997; Chambers & Potenza, 2003). Therefore, elucidating specific brain regions activated during various aspects of reward processing is essential to understanding, diagnosing, and treating these disorders (Chau et al., 2004; Dichter et al., 2012; Zald & Treadway, 2017), as specific abnormalities in reward processing can be observed in different forms of psychopathology (Zald & Treadway, 2017). Moreover, elucidation of reward dysfunction across a range of diagnostic categories may help to refine the phenotypes of brain structure and function and thus improve the prediction of onset and recovery of these disorders (Baskin-Sommers & Foti, 2015). Furthermore, a better understanding of disorder-specific and/or symptom-specific neural correlates of reward processing will help refine brain-based treatment techniques, such as brain stimulation and neurofeedback, in the management of substance use disorders and other related psychiatric disorders (Dobrossy et al., 2021; Mahoney et al., 2020; Peters et al., 2016). Lastly, elucidation of individualized brain connectome-based symptom profiles will help optimize personalized medicine approaches to treating a range of reward-related disorders (Hollunder et al., 2022).

#### 4.2.5. Limitations and Suggestions

While our study has produced some interesting findings, it has a few major limitations. First of all, the sample size of thirty participants is very small, limiting the statistical power in identifying activation clusters as well as reaching any statistical conclusions. Specifically, a smaller sample size limits the power and confidence to elicit and interpret associations between clusters and behavioral features. Second, the sample consisted of only males, and therefore, the results are not generalizable to both genders. Importantly, the absence of female participants precludes any examination of gender-related effects in reward processing, which are well documented in the literature. Third, the age range (19–38) is wide, and brain development and behavioral characteristics may vary between those in their 20s and 30s. Finally, the current study has only analyzed the win–loss contrast; other relevant contrasts (e.g., larger vs. smaller rewards, risky vs. safe bets, etc.) may also be important aspects of reward processing. Furthermore, only the analyses examining outcome, saliency, magnitude, and probability effects together can reliably identify subtle and relative aspects of reward processing in the complex gambling tasks used in the current study. Therefore, a simple contrast of “win–loss” alone, without analyzing bet amounts and probability conditions, may run the risk of missing the nuances of different aspects of reward processing. We suggest conducting future studies with larger sample sizes consisting of both males and females to enhance the statistical power and generalizability of the findings. Future studies of a similar gambling task should analyze different age cohorts to examine reward processing across different developmental stages. Future studies may also consider examining other contrasts and paradigms of reward processing while also examining different measures (e.g., functional connectivity).

## 5. Conclusions

The current study was designed to elicit the neural substrates underlying the reward evaluations of win versus loss outcomes in a monetary gambling paradigm as well as to understand possible associations of these brain regions with behavioral characteristics such as impulsivity, task performance, and neuropsychological measures. Findings revealed that a set of key brain structures, such as the putamen, caudate nucleus, superior and inferior parietal lobule, angular gyrus, and Rolandic operculum, showed greater activation during the win relative to the loss condition, and most of them were highly correlated with each other. Although the multivariate canonical correlation analyses failed to elicit associations between the anatomical regions and behavioral characteristics, exploratory bivariate correlations were significant with moderate effect sizes. Additionally, some of these reward-related regions showed meaningful associations with specific features of impulsivity, task performance, and neuropsychological measures. However, caution should be exercised with regard to these findings, as the study has major limitations, including a smaller sample size and a lack of female participants, which limit the statistical power and generalizability of the findings. Further studies with larger samples are needed to confirm these preliminary findings.

## Figures and Tables

**Table 1:** Various trial types used in the monetary gambling task.

Trial Type	Chance of Winning	Outcome	Number of Trials	Trial Probability (%)
1	50%	Win	40	16.67%
2	50%	Loss	40	16.67%
3	75%	Win	60	25.00%
4	75%	Loss	20	8.33%
5	25%	Win	20	8.33%
6	25%	Loss	60	25.00%

**Table 2:** Behavioral scores extracted from the performance data of the monetary gambling task.

Variable Name	Variable Description	Min	Max	Mean	SD	SE
Net_Outcome	Total tokens won or lost at the end of the task	0	1790	1187.67	386.00	70.47
Bet50_Prv1Loss	# 50 tokens after a loss during the previous trial	16	90	60.77	14.75	2.69
Bet10_Prv1Loss	# 10 tokens after a loss during the previous trial	21	103	56.13	16.05	2.93
Bet50_Prv2Loss	# 50 tokens after two consecutive losses during the previous trials	6	45	30.83	8.63	1.58
Bet10_Prv2Loss	# 10 tokens after two consecutive losses during the previous trials	12	54	27.93	8.98	1.64
Bet50_Prv3Loss	# 50 tokens after three consecutive losses during the previous trials	3	24	15.03	5.17	0.94
Bet10_Prv3Loss	# 10 tokens after three consecutive losses during the previous trials	4	29	13.73	5.85	1.07
Bet50_Prv2NetLoss	# 50 tokens after the net outcome of loss during the previous two trials	23	99	65.5	16.21	2.96
Bet10_Prv2NetLoss	# 10 tokens after the net outcome of loss during the previous two trials	26	125	60.93	18.00	3.29
Bet50_Prv3NetLoss	# 50 tokens after the net outcome of loss during the previous three trials	11	78	51.33	13.66	2.49
Bet10_Prv3NetLoss	# 10 tokens after the net outcome of loss during the previous three trials	27	90	47.47	12.55	2.29

# Number of bets made with 50 or 10 tokens; Min = Minimum; Max = Maximum; SD = Standard Deviation; SE = Standard Error.

**Table 3:** The data obtained for each subscale and total score of the Barratt Impulsiveness scale.

Variable Name	Variable Description	Min	Max	Mean	SD	SE
BIS_NP	Non-Planning	13	34	19.80	4.61	0.84
BIS_MI	Motor Impulsivity	14	27	19.30	3.28	0.60
BIS_AI	Attentional Impulsivity	8	21	12.57	3.19	0.58
BIS_Tot	Total Impulsivity	39	72	51.67	8.60	1.57

Min = Minimum; Max = Maximum; SD = Standard Deviation; SE = Standard Error.

**Table 4:** The performance scores from the Tower of London test.

Variable Name	Variable Description	Min	Max	Mean	SD	SE
TOL_ExcMovMade	Excess moves made	0.00	29.00	7.83	6.66	1.24
TOL_AvgPicTime	Average pickup time	1.47	5.45	2.81	0.96	0.18
TOL_AvgTotTime	Average total time	2.58	8.80	4.72	1.64	0.30
TOL_TotTrlTime	Total trial time	241.65	788.01	404.24	139.05	25.82
TOL_AvgTrlTime	Average trial time	11.51	37.52	19.25	6.62	1.23

Min = Minimum; Max = Maximum; SD = Standard Deviation; SE = Standard Error.

**Table 5:** The performance scores from the visual span test.

Variable Name	Variable Description	Min	Max	Mean	SD	SE
VST_TotCor_Fw	Total correct scores for forward trials	5.00	14.00	10.21	2.78	0.52
VST_Span_Fw	Span for forward trials	4.00	8.00	6.83	1.37	0.25
VST_TotAvgTime_Fw	Total average time for forward trials	9.99	49.11	28.31	10.53	1.96
VST_TotCorAvgTime_Fw	Total correct average time for forward trials	14.90	49.11	32.48	8.07	1.50
VST_TotCor_Bw	Total correct scores for backward trials	5.00	14.00	8.31	1.87	0.35
VST_Span_Bw	Span for backward trials	4.00	8.00	5.52	0.95	0.18
VST_TotAvgTime_Bw	Total average time for backward trials	9.84	56.48	17.79	10.01	1.86
VST_TotCorAvgTime_Bw	Total correct average time for backward trials	14.73	56.48	27.16	10.66	1.98

Min = Minimum; Max = Maximum; SD = Standard Deviation; SE = Standard Error.

**Table 7:** Pearson bivariate correlations between the fMRI activation clusters (C01–C10) and demographic variables (age and education), BIS impulsivity scores, gambling task performance measures, and neuropsychological scores from the TOL test and VST. The significant correlations are highlighted in bold font. The asterisks (* *p* < 0.05; ** *p* < 0.01) represent the level of significance before the Bonferroni corrections. Note that none of the correlations survived Bonferroni corrections, although some of the correlations do have moderate effect sizes.

Variable Set	Variable	C01 R. Ptm	C02 L. Ptm	C03 R. SPL	C04 R. AnGy	C05 L. IOC	C06 R. RoOp	C07 R. Cdt (AI)	C08 R. Cdt (PS)	C09 R. SMG	C10 R. IPL
Demographic variables	Age	−0.1686	−0.0885	−0.3225	−0.0864	0.0366	−0.2015	0.0519	0.1424	0.0452	0.1024
	Education	−0.1406	−0.1360	−0.1668	−0.1591	0.2654	−0.1568	−0.1810	0.0538	−0.1594	0.1576
Impulsivity scores	BIS_NP	−0.3844 *	−0.4057 *	−0.3534	−0.3291	−0.2510	−0.2459	**−0.4072**	−0.5603 **	−0.2798	−0.3155
	BIS_MI	−0.0127	−0.0581	−0.3885 *	−0.0754	−0.2758	−0.2030	−0.1888	−0.0924	−0.0088	0.1499
	BIS_AI	−0.0844	−0.3143	−0.1279	−0.0107	−0.0940	−0.1215	−0.1463	−0.3095	−0.1738	−0.1755
	BIS_Tot	−0.2422	−0.3562	−0.3851 *	−0.2091	−0.2746	−0.2543	−0.3446	−0.4504 *	−0.2178	−0.1770
Task performance	Net_Outcome	−0.1250	−0.2576	0.1526	−0.2325	0.1594	−0.3039	0.0517	−0.0319	−0.2912	0.0202
	Bet50_Prv1Loss	0.3700 *	0.0915	0.1586	0.1642	0.1348	0.3617 *	0.1382	0.0521	0.0873	0.0799
	Bet10_Prv1Loss	−0.2937	−0.1051	−0.0691	−0.0909	−0.2319	−0.1873	−0.1078	0.0133	−0.1279	−0.0318
	Bet50_Prv2Loss	0.3754 *	0.1812	0.2366	0.2337	−0.0326	0.3896 *	0.1347	0.0513	0.1883	0.1301
	Bet10_Prv2Loss	−0.3903 *	−0.1510	−0.0632	−0.0695	−0.1868	−0.2537	−0.1929	−0.0736	−0.2222	−0.0041
	Bet50_Prv3Loss	0.2540	0.1339	0.2415	0.2929	−0.0371	0.3139	0.1563	0.1143	0.2803	0.1552
	Bet10_Prv3Loss	−0.3943 *	−0.0221	0.0013	−0.0020	−0.1044	−0.2551	−0.2749	−0.1478	−0.3456	0.1476
	Bet50_Prv2NetLoss	0.2952	0.0196	−0.0084	0.0484	0.0049	0.3052	0.0501	−0.0094	0.1951	0.0100
	Bet10_Prv2NetLoss	−0.2676	−0.0495	−0.0523	−0.0499	−0.2094	−0.1299	−0.0098	0.1107	−0.0841	0.0173
	Bet50_Prv3NetLoss	0.3246	0.0931	0.0230	0.1136	0.0553	0.3604	0.0022	−0.0122	0.1900	0.0138
	Bet10_Prv3NetLoss	−0.1418	0.0355	0.0016	0.0317	−0.2043	−0.0929	0.0215	0.1228	−0.0544	0.0315
Neuropsychological scores	TOL_ExcMovMade	−0.0468	0.0523	−0.0938	−0.1615	−0.3927 *	0.0679	−0.0179	−0.1394	−0.0035	−0.1466
	TOL_AvgPicTime	0.0411	0.1848	0.2992	0.1470	0.2767	−0.1186	0.1002	0.1636	0.1843	0.0613
	TOL_AvgTotTime	0.0533	0.1626	0.1840	0.0473	0.2300	−0.2029	0.0916	0.1049	0.0622	−0.0133
	TOL_TotTrlTime	0.0514	0.1852	0.1691	0.0168	0.1580	−0.1979	0.0893	0.0756	0.0297	−0.0447
	TOL_AvgTrlTime	0.0514	0.1852	0.1691	0.0168	0.1580	−0.1979	0.0894	0.0756	0.0297	−0.0447
	VST_TotCor_Fw	−0.3981 *	−0.2994	−0.0212	−0.1768	0.1453	−0.1415	−0.1193	−0.0483	0.0324	−0.0508
	VST_Span_Fw	−0.3102	−0.2193	0.0114	0.0489	0.0826	0.0098	−0.0375	0.0441	0.1885	0.0089
	VST_TotAvgTime_Fw	−0.1477	−0.0876	0.0234	0.0592	0.1884	−0.0288	0.1645	0.1863	0.2613	−0.0152
	VST_TotCorAvgTime_Fw	−0.1073	0.0129	0.0125	0.0592	0.1609	−0.0083	0.1710	0.1496	0.2279	0.0012
	VST_TotCor_Bw	0.0308	0.0717	0.2810	0.0232	−0.0563	0.0489	0.2737	0.2256	0.2482	−0.0321
	VST_Span_Bw	0.0492	−0.0505	0.1533	−0.1092	−0.0339	−0.0772	0.2028	0.1715	0.1973	−0.0333
	VST_TotAvgTime_Bw	0.1166	0.0429	0.1718	−0.0298	−0.2722	0.0345	0.2830	0.2251	0.2971	−0.1202
	VST_TotCorAvgTime_Bw	0.1548	0.0176	0.2422	−0.0960	−0.1317	−0.0426	0.2714	0.1209	0.3142	−0.0829

## Data Availability

The data presented in this study will be made available to the researchers upon request to the corresponding author.

## Abbreviations

The following abbreviations are used in this manuscript:

MGT	Monetary Gambling Task
BOLD	Blood Oxygenation Level-Dependent
MRI	Magnetic Resonance Imaging
fMRI	Functional Magnetic Resonance Imaging
BIS	Barratt Impulsiveness Scale
TOL	Tower of London Test
VST	Visual Span Test
MPRAGE	Magnetization-Prepared Rapid Gradient Echo
ART	Automatic Registration Toolbox
sPCA	Sparse Principal Component Analysis
